# Efficacy of a Carrageenan nasal spray in patients with common cold: a randomized controlled trial

**DOI:** 10.1186/1465-9921-14-124

**Published:** 2013-11-13

**Authors:** Martin Ludwig, Elisabeth Enzenhofer, Sven Schneider, Margit Rauch, Angelika Bodenteich, Kurt Neumann, Eva Prieschl-Grassauer, Andreas Grassauer, Thomas Lion, Christian A Mueller

**Affiliations:** 1 Department of Otorhinolaryngology, Medical University of Vienna, Waehringer Guertel 18-20, 1090 Vienna, Austria; 2 Children’s Cancer Research Institute and LabDia Labordiagnostik, Zimmermannplatz 8, 1090 Vienna, Austria; 3 Marinomed Biotechnologie GmbH, Veterinaerplatz 1, 1210 Vienna, Austria; 4 E. I. S. Executive Information Service GmbH, Mariahilfer Straße 88a/1/5, 1070 Vienna, Austria; 5 Department of Pediatrics, Medical University of Vienna, Waehringer Guertel, 18-20, 1090 Vienna, Austria

**Keywords:** Common cold, Virus, Respiratory, Disease, Carrageenan

## Abstract

**Background:**

The common cold is the most widespread viral infection in humans. Iota-carrageenan has previously shown antiviral effectiveness against cold viruses in clinical trials. This study investigated the efficacy of a carrageenan-containing nasal spray on the duration of the common cold and nasal fluid viral load in adult patients.

**Methods:**

In a randomized, double-blind, placebo-controlled trial, 211 patients suffering from early symptoms of the common cold were treated for seven days. Application was performed three times daily with either a carrageenan-supplemented nasal spray or saline solution as placebo with an overall observation period of 21 days. The primary endpoint was the duration of disease defined as the time until the last day with symptoms followed by all other days in the study period without symptoms. During the study, but prior unblinding, the definition of disease duration was adapted from the original protocol that defines disease duration as the time period of symptoms followed by 48 hours without symptoms.

**Results:**

In patients showing a laboratory-confirmed cold virus infection and adherence to the protocol, alleviation of symptoms was 2.1 days faster in the carrageenan group in comparison to placebo (p = 0.037). The primary endpoint that had been prespecified but was changed before unblinding was not met. Viral titers in nasal fluids showed a significantly greater decrease in carrageenan patients in the intention-to-treat population (p = 0.024) and in the per protocol population (p = 0.018) between days 1 and 3/4.

**Conclusions:**

In adults with common cold virus infections, direct local administration of carrageenan with nasal sprays reduced the duration of cold symptoms. A significant reduction of viral load in the nasal wash fluids of patients confirmed similar findings from earlier trials in children and adults.

**Trial registration:**

Current Controlled Trials ISRCTN80148028

## Background

Acute viral respiratory tract infection (ARTI), also known as the common cold, is the most prevalent disease in humans. In the USA alone, non-influenza colds annually account for more than 20 million doctor visits and 40 million lost school and work days. With a total economic impact of approximately $40 billion, ARTI is among the ten most expensive illnesses in society [1].

In the majority of cases, common colds are caused by respiratory viruses such as rhinovirus, coronavirus, parainfluenza, influenza, respiratory syncytial virus, adenovirus, enterovirus, or metapneumovirus [2-7].

The number of etiological agents and antigenic variability has limited the possibility of creating an effective vaccine against the common cold. Numerous attempts to find effective prophylactic or therapeutic treatments including pharmaceutical [8] and herbal [9,10] products, vitamins [11-14], zinc [15] and others have provided controversial results and are not considered effective when systematically reviewed [16-21].

Presently, therapy for the common cold mainly includes general care and treatment of symptoms. While these measures reduce symptoms, therapeutic interventions to date have not been proven to be effective in reducing viral load or the duration or severity of common colds [22].

The concept of using sulphated polysaccharides as antiviral agents was introduced more than 25 years ago [23]. Due to the high molecular weight of polymers, parenteral administration is not feasible. For the treatment of sexually transmitted diseases, research has focused on topical microbicide development although not enough evidence has accumulated to recommend these agents at present. However, these polysaccharides have been tested to prevent sexually transmitted viral infections as a component of spermicides [24,25].

Recently it was shown that iota-carrageenan, a sulphated polysaccharide found in some species of red seaweed, is a potent antiviral agent against respiratory viruses in cell culture and animal models [26,27]. Laboratory data show that the carrageenan-polymer binds directly to the virus but not to cells trapping the particle and preventing cell attachment. Thus, carrageenan interferes with the virus life cycle at a very early stage due to a physical mechanism of action. Studies *in vitro* and *in vivo* have shown the effectiveness of carrageenan against several viruses such as hRV [27], influenza A [26] and RSV. Tests *in vitro* have shown that carrageenan does not penetrate the mucosa. Therefore, pharmacological, immunological or metabolic activities of carrageenan are not to be expected.

In addition to preclinical studies, symptomatic benefit and antiviral efficacy of a nasal spray containing carrageenan has been shown in two randomized clinical trials in adult [28] and pediatric patients with the common cold [29]. This study was designed to investigate the efficacy of a carrageenan-containing nasal spray on the duration of the common cold and the viral load in nasal fluid in adult patients.

## Methods

### Study subjects

All patients were recruited at the Department of Otorhinolaryngology of the Vienna General Hospital, Austria between January, 2010 and April, 2011. Eligible patients were adults (18 years and older) with early symptoms of the common cold (onset less than 48 hours before inclusion) of mild to moderate intensity (Total Symptoms Score [TSS], of 2 to 9).

The intensity of common cold symptoms was assessed according to a published scoring system [30]. Patients scored eight main symptoms (headache, muscle ache, chilliness, sore throat, blocked nose, runny nose, coughing and sneezing) using a four point scale as follows: 0 (symptom absent) and 1, 2 and 3 representing mild, moderate and severe intensity, respectively. TSS was calculated by investigators as the sum of individual scores.

Main exclusion criteria were known hypersensitivity or allergy to any component of the test product, concomitant disease or infection that could interfere with participation in the study, other reasons for nasal obstruction and other past or present conditions and treatments that could influence symptom scores. Specifically, during the treatment period (days 1–7), use of any other nasal spray, nonsteroidal anti-inflammatory drugs, antihistamines, decongestants, corticosteroids, antivirals, antitussives, herbal combinations for common colds and supplements containing ≥10 mg zinc or ≥100 mg vitamin C were prohibited.

The study was performed in compliance with the ICH E6 Note for Guidance on Good Clinical Practices (CPMP/ ICH/ 135/ 95/ 5) and the principles of the Declaration of Helsinki. The Ethics Committee of the participating site approved the protocol and all amendments. Written informed consent was obtained from each participant before enrolment into the study. Participants were compensated for expenses associated with participation.

### Study design

This was a single-center, double-blind, parallel-group, placebo-controlled, randomized (1:1) clinical trial to show differences between carrageenan-containing nasal spray over placebo in adult patients with early symptoms of the common cold.

The objective of this trial was to obtain information on the efficacy of carrageenan-containing nasal spray (Coldamaris prophylactic) in adult patients with early symptoms of the common cold. The aim was to show at least 2 days reduction in the duration of disease with a standard deviation (SD) of 4.9. This resulted in a sample size of 96 per group. With a maximal drop-out rate expected of 25%, the sample size was set at 125 patients per group. Type-I error probability was set to a two-sided alpha of 0.05. Power was set to 80 percent.

### Study procedures

All patients were randomly assigned to receive either Coldamaris prophylactic nasal spray containing carrageenan (0.12%) or saline solution (sodium chloride 9.0 g/l) nasal spray, which served as a placebo. Nasal spray was administered three times daily into each nostril for 7 days. Coldamaris prophylactic nasal spray is a registered medical device marketed in Austria and manufactured by MoNo chem GmbH, Austria.

Each subject was randomised to one of the two groups according to a randomization schedule using random permuted blocks (block size of 4). The randomization list was prepared by a third party and was unblinded after trial review and data base lock.

Each nasal spray had a three-digit code. The sprays were assigned by investigators consecutively starting with the lowest treatment number available. Both investigators and patients were blinded to study treatment. Carrageenan and placebo nasal sprays were identical in shape, size and colour. Both spray solutions were clear, colourless, odourless and free of particulates.

For all enrolled subjects, medical histories were evaluated at baseline. Clinical assessments including ear, nose and throat examination were performed and nasal wash fluid for laboratory testing (viral assessment) was obtained. ENT examinations and nasal wash fluid collection were repeated on days 3/4 (visit 2) and 10/11 (visit 3). During visits, safety evaluation was also conducted. On the last visit (days 22–28), in addition to final safety assessments, all subjects were questioned regarding their opinion as to the acceptability of the study treatment.

During days 1 to 9, patients had to record whether they used the nasal spray as instructed, the presence of any additional symptoms, the use of any additional treatments and scores for the 8 main common cold symptoms described above. This diary had to be returned to the study site at visit 3.

From days 10 to 21, patients kept diaries on a daily basis and recorded common cold symptoms experienced and the product and dosage of any other treatments taken.

Compliance with study treatment was assessed by measuring the weight of the dispensed nasal spray on the days of issuance and return. A difference of less than 4.0 g was assessed as consumption less than 80% of study protocol and such patients were subsequently excluded from PP analysis.

### Viral status assessment

Nasal wash fluid samples were obtained for virological analysis at baseline (day 1), at visit 2 (study days 3/4) and visit 3 (study days 10/11) using the following procedure. Approximately 2.5 ml of 0.9% saline solution were instilled into each nostril and kept in the nose for 10–30 seconds. Patients were then required to lean the head forward allowing saline to drip out of the nose into a collection cup. After collection, mucus was removed and the sample divided into 3 cryovials, labelled and stored at −80°C until assessment. The presence and the amount of viral RNA in nasal wash fluid samples was identified using a real-time, quantitative RT-PCR assay as described previously [29]. Analyses for the respiratory viruses rhinovirus, coronavirus type OC43, coronavirus type 229E, influenza virus type A and B, human metapneumovirus, respiratory syncytial virus and parainfluenza types 1, 2 and 3 were performed.

A patient was considered to be virus-positive when at least one nasal wash fluid sample was tested positive for one virus.

### Endpoints

The primary efficacy variable was the duration of disease defined as the time until the last day with symptoms followed by all other days in the study period without symptoms.

The main secondary efficacy variables were the presence of cold viruses in nasal fluid samples, severity of common cold symptoms on separate study days, number of days without symptoms during the observation period, use of co-medication additional to the study treatment between days 8–21 (after finishing treatment with the carrageenan nasal spray) and the number of cleared or newly acquired viral infections during the observation period.

**Figure 1:**
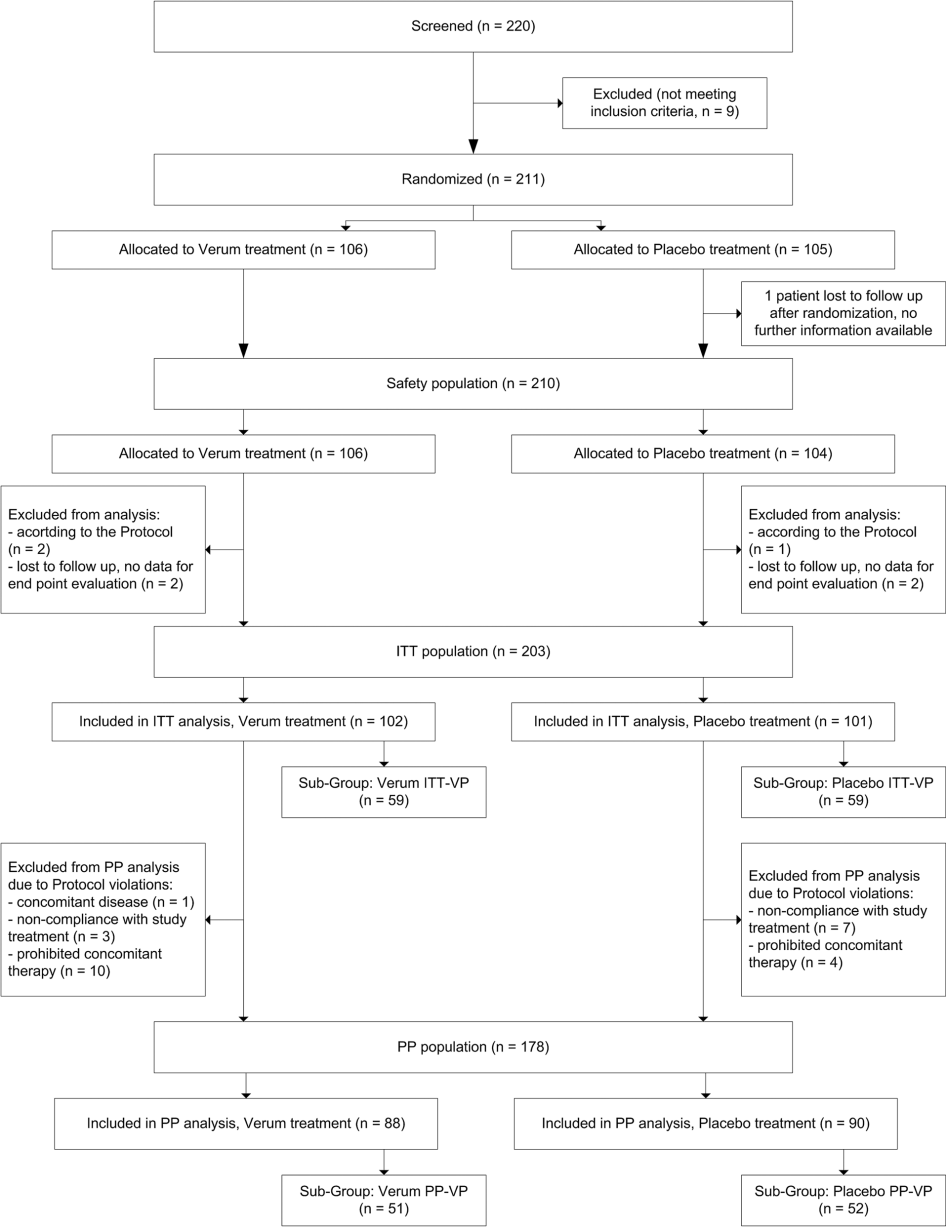
COP 03/09 trial, patient flow chart.

During the course of the study, but prior to unblinding the data and performing analysis, the following efficacy outcomes were adapted based on results obtained in a previous common cold trial. The primary efficacy parameter was changed from “the time period between first and last day with symptoms followed by 48 hours without symptoms” to “the time period between first and last day with symptoms, followed by all other days without symptoms” – a definition that was used in another study of carrageenan in a pediatric population [29]. Due to the physical mechanism of action of the tested polymer, only patients with a cold virus in the nasal cavity are likely to benefit from local antiviral treatment with iota-carrageenan. Hence, analysis of effectiveness was carried out for patients with laboratory confirmed cold virus infection (Figure 1). Therefore, the intention-to-treat virus positive population (ITT-VP) as well as the per protocol virus positive population (PP-VP) were assessed. For analysis of safety data all patients who received a study medication were included (ITT). The protocols, all amendments including those to the primary endpoint and the assessment of the virus positive population were approved by the local ethics committee prior unblinding of the study.

### Statistical analysis

The primary end point was analysed using the log rank test and the Mantel-Haenzel test. All data sets were checked for normal distribution by the Kolmogorov-Smirnov-Test prior to analysis. Binary or categorical data (e.g. presence or absence of viruses, number of patients with adverse events [AE]) were analysed by the Chi-square test.

Viral titer in nasal wash fluids as well as changes in titers between visits were analysed using the median test due to high variability. In the dataset for this analysis, all viruses detected were included and each virus was accounted for individually. Since some patients had more than one virus, the number of viral events was higher than the number of infected patients.

Experience from previous trials suggested that TSS data might not be normally distributed and lopsided. This was confirmed for the actual study with a Kolmogorov-Smirnov test and a test for lopsided distribution of data. Hence analysis of TSS with standard conventional methods was not applicable. Prior to unblinding, it was decided to apply the analysis of ReWuMoD (regression with unknown model domain, Additional file 1: Figure S1) technique instead of conventional statistical methods. The principles of this technique have been described in detail previously [31]. Briefly, this model transformed 10 TSS values (baseline and days 1–9) into a two line regression model for each individual patient. The average parameters of these two lines allowed the comparison of the course of the disease between the treatment groups irrespective of absolute TSS values.

ReWuMoD analysis yields a reduction from ten individual data points (TSS values) to five relevant parameters. Intercept and slope of the first straight line (with the parameters a1 and b1; representing the course of the early phase of the disease), the estimated/calculated break point (BP) between the two lines (representing the time point of the change in the course of the disease) and the parameters of the second line (abbreviated as a2 and b2; representing the course of the later phase of the disease).

It was further reasoned that the ReWuMoD model is less sensitive to absolute score values allowing the evaluation of different courses of disease independent of absolute values.

### Safety

All findings after the screening visit were documented as AEs except for any symptoms attributed to common cold (sneezing, runny nose, nasal obstruction, sore throat, cough, headache, fatigue and chilliness) as defined in the protocol. Nevertheless, if such symptoms were of marked intensity or deemed to be not related to the study disease, these were recorded as AEs.

## Results

### Patients

Altogether, 211 patients with suspected common cold were randomized with 203 completing treatment in the ITT population. Reasons for patient exclusion were lost to follow-up (5 patients) and exclusion according to protocol (3 patients). None of the patients discontinued treatment due to adverse events (Figure 1).

The PP population consisted of 178 patients. Main reasons for exclusion were the use of prohibited medications (n = 14) and non-compliance (n = 10). Additionally, one patient was enrolled with violation of inclusion/exclusion criteria (chronic obstructive pulmonary disease as concomitant disease).

The virus positive populations consisted of 118 patients in the ITT-VP and 103 patients in PP-VP groups (Figure 1).

The ITT population comprised 110 females (59 placebo and 51 carrageenan) and 93 males (42 placebo and 51 carrageenan). The mean age was 33.3 years in the placebo and 33.7 years in the carrageenan group.

There were no statistically significant differences in demographic variables, smoking status and intensity of symptoms between treatment groups in the ITT/ITT-VP (Table 1) or PP/PP-VP (data not shown) populations.

**Table 1:** Demographic and baseline characteristics, ITT and ITT-VP population

	**ITT**					**ITT-VP**				
	**Carrageenan**		**Placebo**			**Carrageenan**		**Placebo**		
	**n**	**%**	**n**	**%**	**p value**	**n**	**%**	**n**	**%**	**p value**
Gender										
Male	51	50.0%	42	41.6%		29	49.2%	24	40.7%	
Female	51	50.0%	59	58.4%	0.23*	30	50.8%	35	59.3%	0.36*
	Mean	SD	Mean	SD		Mean	SD	Mean	SD	
Age, years	33.7	12.9	33.3	12.9	0.84**	31.6	12.0	32.6	12.9	0.65**
Weight, kg	74.0	15.4	73.8	15.7	0.95**	71.5	13.1	70.1	13.8	0.56**
Height, cm	174.0	10.4	173.2	9.4	0.55**	174.3	10.1	172.8	9.9	0.86**
	n	%	n	%		n	%	n	%	
Smoking status										
Active smoker	36	35.3%	32	31.7%	0.59*	24	40.7%	17	28.8%	0.18*
Passive smoker	25	24.5%	22	21.8%	0.65*	17	28.8%	13	22.0%	0.40*
Active OR passive smoker	44	43.1%	40	39.6%	0.61*	30	50.8%	21	35.6%	0.09*
	Mean	SD	Mean	SD		Mean	SD	Mean	SD	
Baseline TSS	6.3	2.0	6.4	1.9	0.56**	6.3	2.0	6.4	1.7	0.77**

*Chi-square test.

**t-test.

### Virus identification

In total, 118/203 (58.1%) patients in the ITT population had a laboratory confirmed infection with at least one common cold virus, with no significant difference in virus distribution between the groups. Total numbers of virus-positive patients per treatment group as well as numbers of patients infected with specific viruses are shown in Table 2. Influenza virus B, and parainfluenza virus types 1 and 2 were not detected in any of patients. 15% of patients tested positive for more than one virus in the nasal fluid.

**Table 2:** Number of virus-positive patients and virus distribution between treatment groups*

	n VP patients*	**RSV**	**Corona 229E**	**Corona OC43**	**hMPV**	**hRV**	**Influenza A**	**PIV3**
**ITT**	118/203	4	32	21	5	60	13	1
**V**	59/102	3	16	13	2	27	7	0
**P**	59/101	1	16	8	3	33	6	1

*Some patients had more than one virus.

hMPV = Human metapneumovirus; hRV = Human rhinovirus; ITT = Intention to treat population; P = Placebo; PIV3 = Parainfluenza virus type 3; V = Carrageenan; VP = Virus-positive patients.

### Primary endpoint: duration of disease

**Figure 2:**
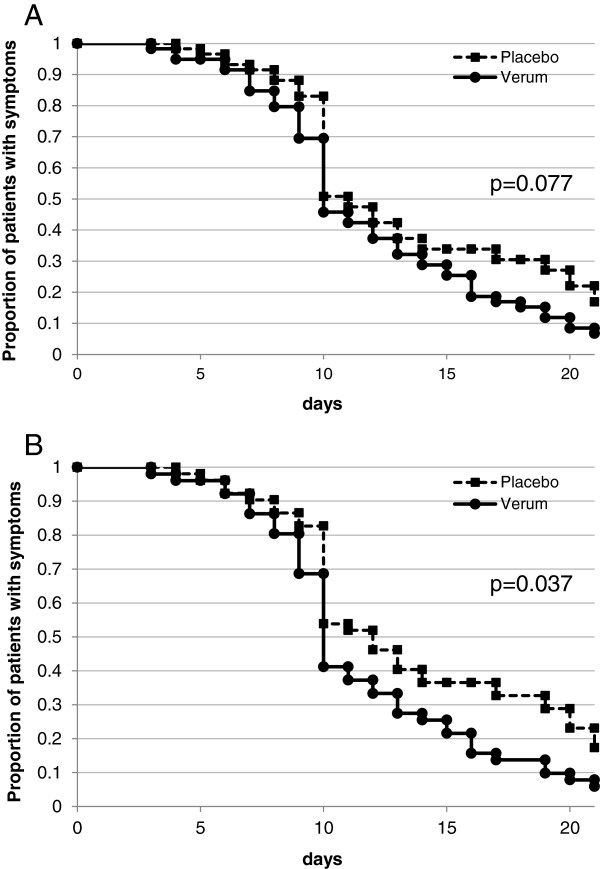
**Alleviation of symptoms in patients infected with a cold virus treated either with intranasal Carrageenan or placebo.** Alleviation was defined as the time until complete absence of cold symptoms followed by all other days in the study period without cold symptoms. Figure 2**A** shows the ITT-VP population p = 0.077 and Figure 2**B** shows the PP-VP population p = 0.037.

In patients with a laboratory confirmed cold virus infection, analysis with a log-rank test showed faster recovery in iota-carrageenan treated patients compared to placebo in the PP-VP population with an estimated duration of disease of 11.6 days (95% confidence interval [CI]; 10.4-12.9 days) in the carrageenan group compared to 13.7 days (95% CI; 12.2-15.1 days) in the placebo group (log-rank test, p = 0.037, Figure 2B).

The relative risk for iota-carrageenan treated PP-VP patients to reduce the symptoms for 1 day was 1.24 (95% CI; 0.88-1.73). The absolute risk reduction (ARR) was −0.13 (95% CI; -0.32-0.08) and the number needed to treat (NNT) was 7.8 (95% CI; 3.1-infinite).

A similar trend was observed in the ITT-VP population, however, the difference was not statistically significant (log-rank test, p = 0.077, Figure 2A). The applied log rank test is more sensitive when the ratio of hazards is higher at early survival times, which is clearly not the case in this study. Further analysis with the Mantel-Haenszel test revealed slightly different p-values but did not change the overall result (ITT-VP: p = 0.051, PP-VP p = 0.020). The relative risk for iota-carrageenan treated ITT-VP patients to reduce the symptoms for 1 day was 1.09 (95% CI; 0.79-1.49). The ARR was −0.051 (95% CI; -0.24-0.14) and the number needed to treat NNT was 19.6 (95% CI; 4.1-infinite).

In the ITT population (N = 203) no statistical significance for the duration of disease was observed.

### Symptoms scores

**Figure 3:**
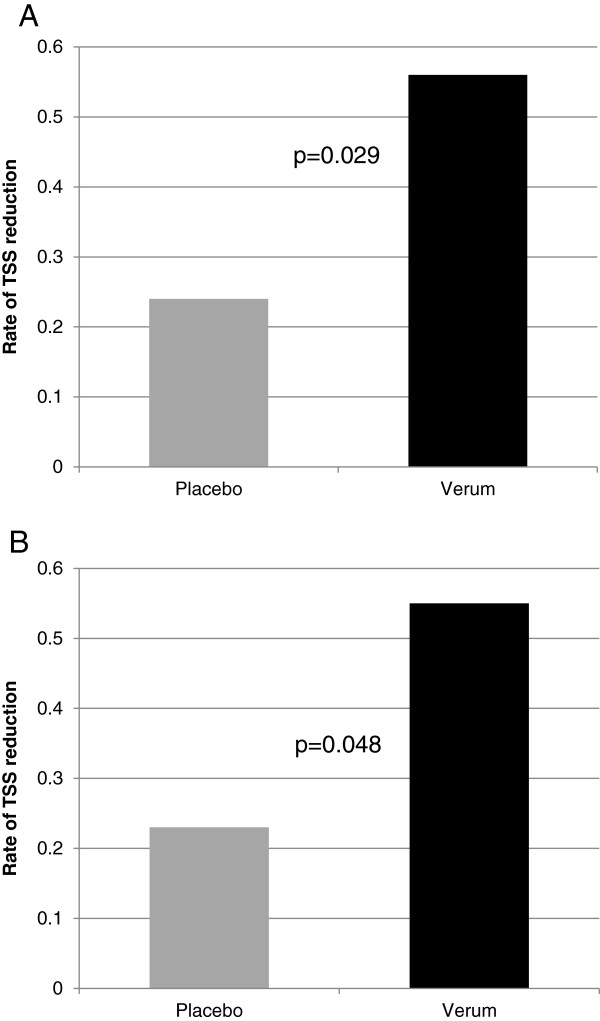
**Mean daily rate of TSS reduction in the second phase of the cold.** The mean daily rate of TSS reduction was calculated from the variable a2 that was determined by ReWuMod analysis and is shown for the ITT-VP **(3A)**, p = 0.029 and PP-VP populations **(3B)** p = 0.048. Details describing the ReWuMod analysis are shown in supplementary Figure 1.

Analysis of TSS using ReWuMoD in virus-positive populations (ITT-VP and PP-VP) did not show statistically significant differences between groups during the early phase of the disease. However, in the ITT-VP population the break point was achieved in the carrageenan group 0.5 days earlier (mean 3.4 days in the carrageenan arm compared to a mean 3.9 days in the placebo arm, p = 0.025, possibly representing an earlier start of the recovery phase), followed by a significant faster reduction of symptom intensity in the carrageenan group (mean TSS decrease 0.56 points/day in the carrageenan group compared to a mean 0.24 points/day in the placebo group, p = 0.029, Figure 3A) in the second phase of the disease. Similar results were also observed in PP-VP patients. The break point was observed earlier in the carrageenan group (mean 3.4 days in carrageenan compared to a mean of 3.9 days in placebo, [p = 0.064]), and this was followed by a faster decrease in TSS during the second phase of the disease. The mean TSS decreased 0.55 points/day in the carrageenan group compared to 0.23 points/day in the placebo group (p = 0.048, Figure 3B). In the ITT (N = 203) no difference for the breakpoint was observed. In the second phase of the disease TSS decreased 0.58 points/day in the carrageenan group compared to 0.34 points/day in the placebo group (p = 0.045).

A posthoc analysis of symptom data revealed that in the ITT-VP population, 39 patients (33%) had re-appearing symptoms (25 placebo patients and 14 carrageenan patients). This difference between the groups was significant with a p-value of 0.031.

### Use of additional treatments

Overall, the number of patients taking medications for common cold symptoms was low (less than 17% in total) with 15 patients in the carrageenan group and 19 patients in the placebo group taking additional medications (ITT) during the study period. In the PP population, 6 patients in the carrageenan group compared to 14 patients in the placebo group consumed additional medication. The difference between the groups represents a trend but was not statistically significant (p = 0.065).

### Viral load in nasal fluid

**Figure 4:**
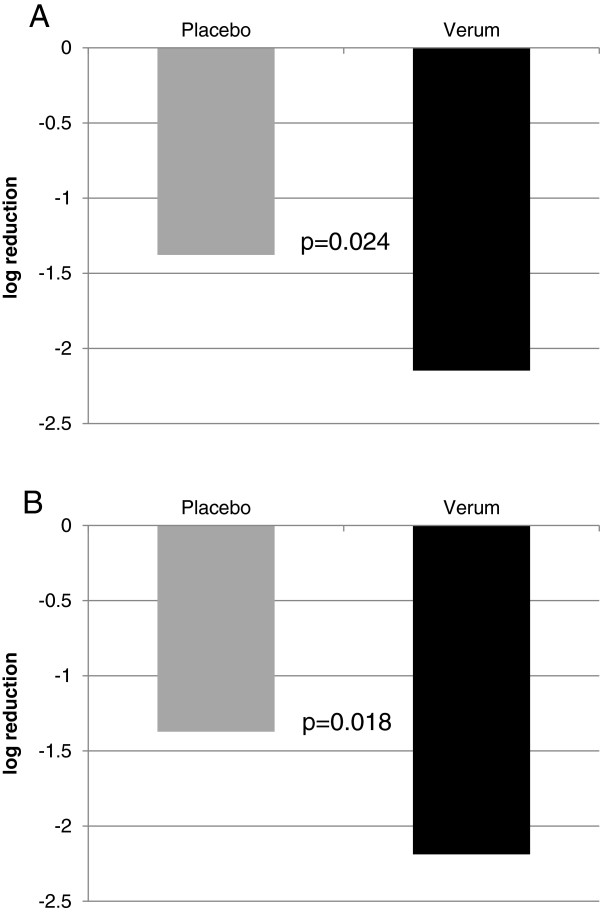
**Differences in viral load between days 1 and 3/4.** The median log_10_ difference of the viral load between days 1 and days 3 or 4 determined with quantitative real time PCR for a set of respiratory viruses as described in the materials and methods is shown. The titers were compared by applying a Median-test. **A**: ITT-VP p = 0.024; **B**: PP-VP: p = 0.018. The black bar shows Iota-Carrageenan and the grey bar shows placebo.

In the nasal fluid of both carrageenan and placebo patients, viral titers decreased from visit 1 to visit 2. However, carrageenan treated patients showed a significantly greater decrease in viral titer between visit 1 and visit 2 (Figure 4). In the ITT-VP population, the median decrease of the log_10_ viral titer was 2.15 in the carrageenan group compared to 1.38 in the placebo group (p = 0.024, Figure 4A). In the PP-VP population, these figures were 2.19 and 1.37 for the carrageenan and placebo groups, respectively (p = 0.018; Figure 4B). Median titers at visit 3 (day 10) were 0 for all patient groups and no further analysis was performed.

### Safety

Overall, the study treatment was well tolerated and no statistically significant differences were found between the study groups regarding the total number or distribution of AEs. In total, 43 AEs (related or not related) were reported, involving 38 subjects (19 subjects in each treatment group) in the trial. There was no significant difference in the frequency of AEs between treatment groups, with the majority of AEs observed only once or twice per SOC (System Organ Class) category (corresponding to a frequency of 0.94% - 1.92%).

Only one AE in the placebo group (the feeling of burning in the nose) was considered to be related to study treatment by the investigator. The vast majority of AEs (41 of 43) were of mild intensity. One AE in the carrageenan group (cycling accident with cerebral commotion and subarachnoidal hemorrhage) was of moderate intensity and one AE in the placebo group (gastric hypermotility) was rated as severe.

In this study, 3 severe AEs in 2 patients (not related and resolved by the end of the study) were reported in the carrageenan group. None of the patients discontinued treatment or participation in the trial due to an AE.

## Discussion

This double blind placebo controlled trial evaluated the effectiveness of a carrageenan nasal spray for the treatment of adult patients with common cold symptoms. This study confirms the results of earlier studies in adults and children. For patients compliant to the protocol and positive for a cold virus infection, a reduction of disease duration of 2.1 days was observed. The results therefore confirm that the local antiviral effectiveness of carrageenan leads to a significant clinical benefit. A pronounced trend was also observed in the ITT-VP population although differences did not reach the level of statistical significance. The main difference between the ITT-VP and PP-VP populations is the use of concomitant medication during the study. Patients were excluded from the PP analysis due to insufficient use of study medication when a reduction of dosage of more than 20% corresponding to 1.5 days of treatment was documented. Thus it can be concluded that tested dosage of 3 times per day is necessary and fewer applications may lead to a reduced clinical benefit.

As mentioned in the materials and methods section the primary endpoint was changed during the course of the study, prior unblinding and with approval of the ethics committee based on results of an earlier study in children. Analysis of the duration of disease according to the endpoint “time period between first and last day with symptoms followed by 48 hours without symptoms” did not result in significant difference in any analysed population neither in the adults or children. Hence, it can be concluded that a part of the positive effect of iota-carrageenan treatment is based on the ability to prevent the reappearance of symptoms when there is a symptom free period longer than 48 hours.

The results reported are limited to patients tested positive with real-time PCR, which is 58% of the study population. Analysis of the primary endpoint for the complete study population (ITT, N = 203) did not reveal a significant difference between the iota-carrageenan and the placebo group. However, analysis of TSS using ReWuMoD revealed a significant faster reduction of symptoms in the second phase of the disease. Similar to the ITT-VP group the reduction of symptoms in the iota-carrageenan group was approximately doubled when compared with the placebo group. This result indicates that independent of the actual cause of symptoms, iota-carrageenan treatment results in a faster reduction of symptom severity.

Due to the physical mechanism of action of carrageenan, it is unlikely that patients with cold symptoms not caused by a virus would have an added benefit in comparison to placebo treatment with a saline nasal solution. Treatment of common colds with saline nasal irrigation has been discussed to have a clinical benefit and has been described to be associated with less time off work [32]. Consequently, the current study is limited by the fact that the placebo nasal spray containing a saline nasal solution most likely has caused a clinical benefit as well. This could explain why no significant effect on the primary endpoint in the ITT population was observed in this trial in contrast to the virus positive population that comprised 58% virus positive patients.

A recent study in children with a similar design revealed a significant reduction of time to disease clearance of 1.8 days in the ITT population comparable to this study [29]. In that children trial around 90% of the children were tested virus-positive and hence there was negligible difference between the ITT and the ITT-VP population, although also in this study the ITT-VP population experienced a stronger effect of treatment with carrageenan. It can be assumed that both, adults and children infected with common cold viruses will benefit from local application of carrageenan in a similar way. In adults, the effect on disease duration might be masked by patients with cold symptoms not caused by viral infections. However, the faster rate of symptom reduction in all iota-carrageenan treated adults suggests that all patients with cold symptoms will have an added benefit when compared with a standard saline nasal spray that served as a placebo in the trial.

Analysis of TSS showed faster symptom improvement in carrageenan treated patients during the second phase of disease with statistically significant differences in the ITT-VP and PP-VP groups. In addition, the break point (representing changes in course of disease and start of recovery) was earlier in the ITT-VP and PP-VP in carrageenan patients. The applied ReWuMod method allows the evaluation of different courses of disease independent of absolute values. This was of particular interest as absolute values depend on different pathogens and subjective patient assessment. No significant difference in TSS between carrageenan and placebo groups was observed in the first 3 days of the study. However, carrageenan treated patients experienced a significantly earlier improvement in disease symptoms. Furthermore, the rate of symptom reduction per day was approximately doubled. It can therefore be concluded that the significant reduction in time to disease clearance was achieved by a faster decrease of symptoms visible on day 4 after inclusion.

The results are in line with data from a trial conducted in adults that showed a significant benefit on TSS in a relatively small group of patients [28]. The trial conducted by Eccles et al. showed that within the first two days after inclusion, patients did not report an improvement in symptom scores. However, starting on day 3, carrageenan treated patients reported a greater reduction in TSS than placebo patients, resulting in a significant clinical benefit and a significant reduction in TSS on day 3. The clinical relevance of this finding is supported by the fact that symptom scores on day 3 of carrageenan treated patients were at the same levels as placebo treated patients on day 5, indicating a reduction in time to symptom alleviation of 2 days. This compares well with the 2.1 day reduction obtained in this trial.

Similar to all studies with the natural common cold, this trial is limited by an uncertainty over the actual dates and times of disease onset. There is a potential for inclusion of patients at later stages of disease which could be a source of bias diminishing treatment efficacy in natural settings. Comparing results from studies of natural and experimental colds suggested that a substantial proportion of patients with natural colds suffered from symptoms up to 1 day longer than they reported [33]. Furthermore, in one study analysis of patient records revealed that up to 35% of patients with a natural cold, who reported being sick for up to 48 hours (inclusion criteria), were in fact ill for 3 days and more [34]. As in any antimicrobial or antiviral intervention, an early start of treatment with carrageenan is likely to provide the best benefit for patients.

The rate of exposure of cigarette smoke in this trial was high (Table 1) Emerging evidence suggests that cigarette smoke affects viral and bacterial binding to epithelial cells [35,36]. A post hoc analysis was conducted to reveal whether iota-carrageenan treatment is beneficial for patients exposed to cigarette smoke. Iota-carrageenan treated patients in the ITT-VP smoke exposed population (includes smokers and smoking exposed) showed an estimated duration of disease of 10.0 days (95% confidence interval [CI]; 8.1-12.0 days) in the carrageenan group compared to 13.6 days (95% CI; 10.9-16.3 days) in the placebo group (log-rank test, p = 0.031). When compared with the general population it is interesting that smoke exposed iota-carrageenan treated patients had a shorter duration of disease while there was no difference in the placebo group. The trial was neither designed nor powered to demonstrate the effect in this particular subgroup. Hence this encouraging results need to be viewed with caution.

A secondary objective of the study was the determination of viral pathogens and intranasal viral loads. Analysis of nasal fluid samples yielded seven different respiratory viruses (Table 2). As expected in common cold patients, more than 80% of virus positive patients suffered from a rhinovirus or corona virus infection. Viral titers in both carrageenan and placebo treated groups decreased from baseline to the second visit on day 3/4. This result is expected in the natural course of the common cold. However, the magnitude of reduction was greater in patients receiving the carrageenan nasal spray and was statistically significant in the ITT and the PP population. The data correspond to results from two earlier clinical trials in adults and children that both revealed a significant reduction of viral load in the nasal secretion of carrageenan patients [28,29].

In this trial intranasal treatment with the carrageenan nasal spray was safe. No drug related serious adverse events were detected. This is most likely attributed to the physical mechanism of action and the fact that carrageenan is not capable of cell invasion. Due to the presumably very low rate of serious adverse events the study was insufficiently powered to detect them.

## Conclusions

The use of carrageenan nasal spray was associated with a significant reduction of viral load in nasal fluids. Common cold patients with laboratory confirmed cold virus and compliant with study treatment showed shorter duration of disease and faster reduction of common cold symptoms when treated with carrageenan. Furthermore, the use of carrageenan nasal spray was safe and well tolerated. Appropriate dosage seems to be essential and early intervention is suggested to result in significant clinical effectiveness. Therefore, iota-carrageenan may be a good alternative treatment for viral infections of the upper respiratory tract known as the common cold.

## Competing interests

A. Grassauer and E. Prieschl-Grassauer hold management positions in Marinomed Biotechnologie GmbH, the study sponsor.

A. Bodenteich is a full time employee of Marinomed Biotechnologie GmbH.

K. Neumann was compensated for work on statistical analysis by Marinomed Biotechnologie GmbH.

T. Lion received reimbursement for the performance of molecular analysis by Marinomed Biotechnologie GmbH.

## Authors’ contributions

ML performed the study on site and drafted the manuscript. EE and SS participated during data acquisition. MR and TL carried out the molecular analysis. KN participated in the design of the study and performed the statistical analysis. EPG, AG and AB participated in the design and coordination of the study. CAM conceived of the study, and participated in its design and coordination and helped to draft the manuscript. All authors read and approved the final manuscript.

## Supplementary Material

Additional file 1: Figure S1 Description of ReWuMod analysis. a1 = start point of the first regression line (y-axis). b1 = slope of line 1, representing the rate of improvement during the initial phase of the disease. BP = break point between line 1 and 2 in days (x-axis), representing the time point of the change in the course of the disease. a2 = start point of the second regression line (y-axis). b2 = slope of line 2, representing the rate of improvement during the later phase of the disease. Click here for file
