# Supplementary figures and images for: Efficacy of a Carrageenan nasal spray in patients with common cold: a randomized controlled trial
Source: Respir Res. 2013 Nov 13;14(1):124. doi: 10.1186/1465-9921-14-124 (PMC3840586; doi:10.1186/1465-9921-14-124)

## Slide 1
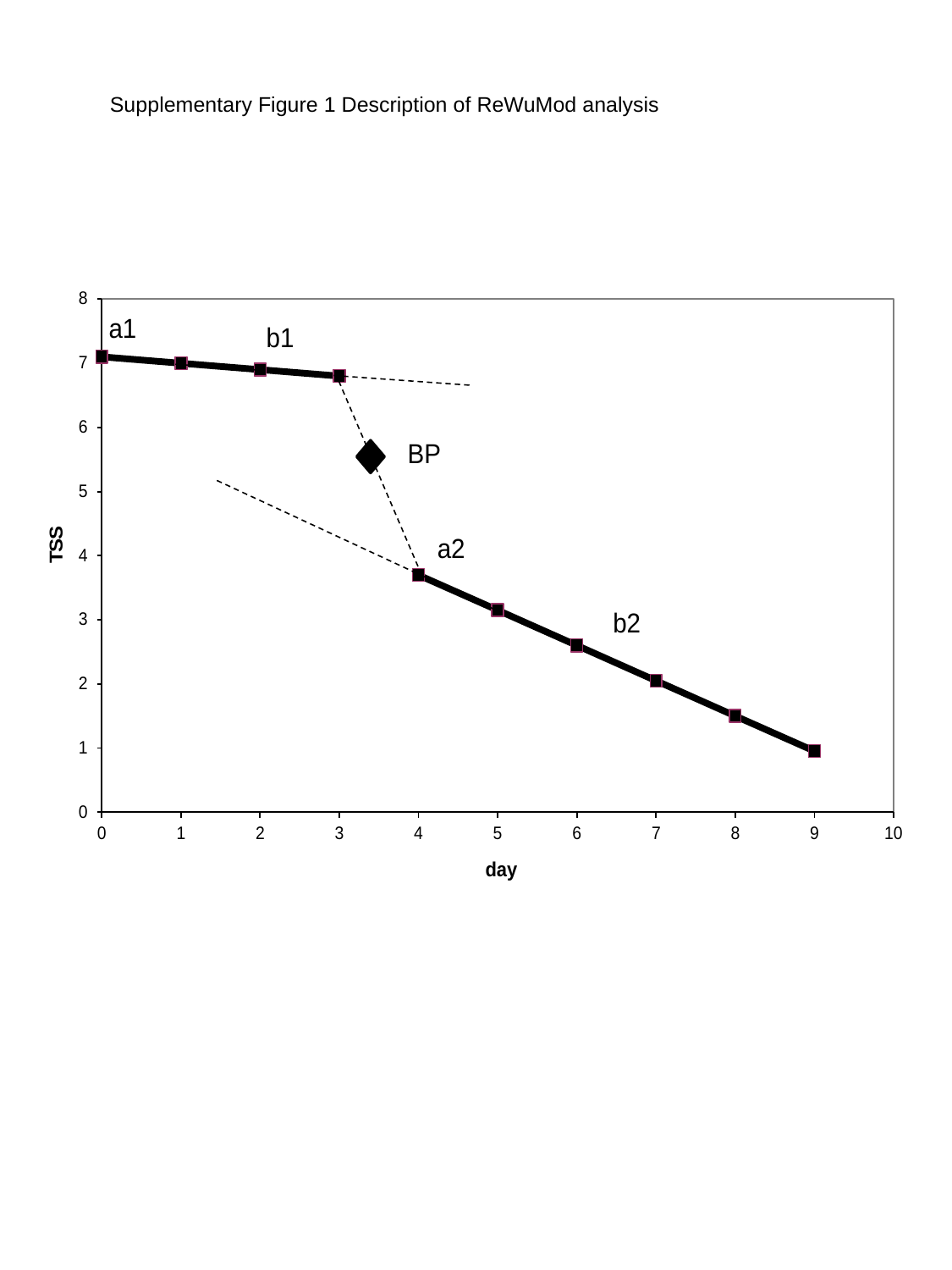

Supplementary Figure 1 Description of ReWuMod analysis

Supplement: Additional file 1: Figure S1 — Description of ReWuMod analysis. a1 = start point of the first regression line (y-axis). b1 = slope of line 1, representing the rate of improvement during the initial phase of the disease. BP = break point between line 1 and 2 in days (x-axis), representing the time point of the change in the course of the disease. a2 = start point of the second regression line (y-axis). b2 = slope of line 2, representing the rate of improvement during the later phase of the disease. [file 1465-9921-14-124-S1.pptx]
